# Quantification of Cisplatin Encapsulated in Nanomedicine: An Overview

**DOI:** 10.3390/bios15050293

**Published:** 2025-05-06

**Authors:** Ziwen Zhang, Jiayu Chen, Tao Wen, Hong Deng, Yiyi Zhang, Hua Guo, Hui Chang, Haiyan Xu, Weiqi Zhang

**Affiliations:** 1 Key Laboratory of Resource Biology and Biotechnology in Western China, College of Life Sciences, Northwest University, Xi’an 710069, China; 2 Institute of Basic Medical Sciences, Chinese Academy of Medical Sciences and Peking Union Medical College, Beijing 100005, China; 3 State Key Laboratory of Molecular Oncology and Department of Molecular Oncology, National Cancer Center/National Clinical Research Center for Cancer/Cancer Hospital, Chinese Academy of Medical Sciences and Peking Union Medical College, Beijing 100021, China

**Keywords:** cisplatin, quantification, nanoparticles, electrochemical sensor, drug delivery

## Abstract

Cisplatin, which kills cancer cells mainly through DNA crosslinking, has been widely used as a first-line chemotherapeutic agent although it also causes severe side effects. To improve anticancer outcomes, various types of cisplatin-based nanomedicines have been developed, either through direct incorporation or coordination of cisplatin within nanoparticles (NPs). During the formulation and characterization of cisplatin-loaded NPs, quantitative determination of cisplatin is crucial for both clinically used and newly developed NPs. While NPs facilitate cisplatin delivery, the use of different nanomaterials inevitably complicates its determination and increases the cost of quantification. Currently, there is still a significant demand for an accurate, simple, and cost-effective method to determine cisplatin in NPs, which would facilitate the screening and quality control of cisplatin-based nanomedicines. This review aims to discuss the main strategies for quantifying cisplatin, following a summary of the main types of cisplatin-loaded NPs. Application examples of cisplatin determination in NPs are provided, and the key features of each quantification strategy are compared. In addition, NP-based electrochemical sensors are included as an emerging approach for characterizing cisplatin loaded in NPs. Rational selection of an appropriate cisplatin determination method for NPs according to the quantification principle and specific drug-delivery settings is highly recommended.

## 1. Introduction

**Figure 1:**
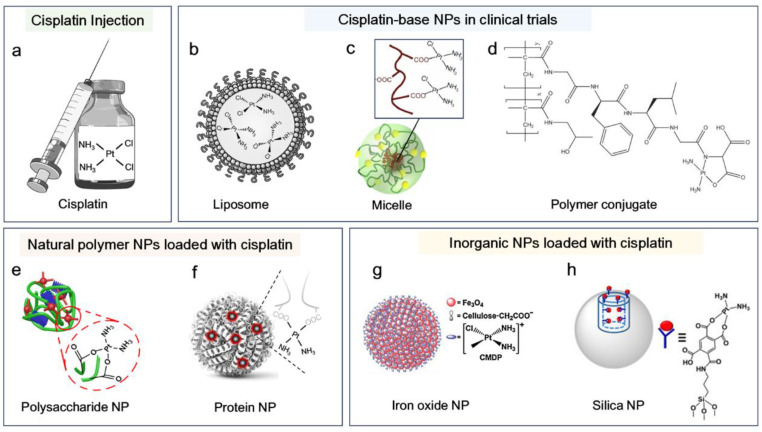
Cisplatin and its representative nanoparticle (NP) formulations. (**a**) Cisplatin structure and clinically used injection solution. (**b**–**d**) Representative cisplatin-loaded NPs that are in clinical trial stages: (**b**) liposomal formulation, e.g., lipoplatin [10]; (**c**) micelle formulation (e.g., NC-6004), adapted from Ref. [11] with permission from Elsevier; (**d**) HPMA-cisplatin conjugate (AP5280), adapted from Ref. [12] with permission from the Royal Society of Chemistry. (**e**,**f**) Cisplatin-loaded NPs composed of natural polymers: (**e**) polysaccharide NP [13]; (**f**) albumin NP [14]. (**g**,**h**) Inorganic NPs with cisplatin encapsulation: (**g**) iron oxide NP, adapted from Ref. [15] with permission from Elsevier; (**h**) silica NP, adapted from Ref. [16] with permission from Elsevier.

Cis-diamminedichloroplatinum (II), also known as cisplatin, is one of the main metal drugs that have been clinically used to treat different types of solid tumor including lung, ovarian, gastric, head, and neck cancers. After almost 50 years since cisplatin’s approval, platinum-based drugs are still among the most used chemotherapeutics, as they are involved in chemotherapies for up to half of cancer patients [1,2]. Cisplatin has the chemical formula cis-[Pt(NH_3_)_2_Cl_2_], and is composed of a central platinum (Pt) atom, two ammonia (NH_3_) groups, and two chloride (Cl) ligands arranged in a square planar geometry (Figure 1a). In aqueous solution, the Cl ligand, as a leaving group, can be easily detached from the Pt core, allowing the Pt to coordinate with nucleophilic sites of biomolecules (e.g., DNA) in cells. This process has been referred to as the aquation of cisplatin because Cl is replaced by water molecules within a low Cl^−^ environment. Within extracellular space, the Cl^−^ is around 100 mM, but it is relatively low in cytoplasm (4–10 mM). Consequently, cisplatin is readily aquated after entering the cytoplasm, becomes activated, and subsequently coordinates with DNA in the nucleus [1,3]. While DNA crosslinking is mainly responsible for chemotoxicity, activated cisplatin can also coordinate with other biomolecules such as proteins, lipids, and saccharides [4,5]. Overall, cisplatin-related cell death results from multiple pharmacological actions within cells. Cisplatin can efficiently diffuse through the cell membrane or be actively imported via membrane transporters such as the copper transport protein CTR1, which lacks selectivity between cancerous and normal cells, thus accounting for the systemic side effects. Although cisplatin is widely used to treat various solid tumors, the clinical application of cisplatin is largely limited by its dose-dependent toxicity. Another challenge for cisplatin-based chemotherapy is the issue of drug resistance, which is caused by multiple mechanisms of action [6]. For example, cisplatin can be exported from cells through ATP-binding cassette (ABC) transporters which are mainly responsible for multi-drug resistance [7]. In addition, cisplatin can also be coordinated with nucleophilic thiol-containing components in cells, e.g., glutathione (GSH) [8,9]. The chelation between cisplatin and GSH is strong, thus deactivating cisplatin, which also contributes to cisplatin resistance.

## 2. Cisplatin-Based Nanomedicine

### 2.1. Nanomedicine-Based Strategies to Enhance Cisplatin Therapy

To overcome the disadvantages of cisplatin treatment, several strategies including new cisplatin analogues and drug delivery systems have been extensively explored [1]. Chemical derivatives of cisplatin such as carboplatin and oxaliplatin have been used in clinical practice; these generate similar anticancer activities but with improved safety performance. Loading cisplatin into NPs provides several therapeutic advantages compared with unencapsulated cisplatin. Cisplatin-loaded NPs can enable controlled drug release and protect cisplatin from undesired interactions with nucleophilic species such as glutathione (GSH) [17,18]. The physicochemical properties of NPs can mediate passive targeting of cancer cells due to the well documented enhanced permeabilization and retention (EPR) effects of solid tumor [19]. Moreover, through further modifying the cisplatin-loaded NPs with targeting ligands such as antibodies, active targeting of cancer cells can also be realized via specific recognition between the targeting ligand and cancer cells. In addition, retention of cisplatin-loaded NP in cells helps avoid the effects of drug efflux, which is a well-known mechanism of cisplatin resistance. Due to the above formulation advantages, cisplatin nanomedicine has been demonstrated to prolong blood circulation, mediate tumor-targeted delivery, reverse drug resistance, and alleviate side effects [20,21]. Consequently, cisplatin-loaded NPs represent one of the widely researched cancer nanomedicines.

### 2.2. Advances in Cisplatin-Based Nanomedicine

To date, different strategies have been employed to construct cisplatin nanomedicine. NPs have been composed using synthetic polymers, natural biomacromolecules, and inorganic materials (Figure 1) [18,22]. Cisplatin is hydrophobic and thus can be easily incorporated into NPs through hydrophobic interaction. The liposomal formulations of cisplatin, e.g., lipoplatin, have entered phase III clinical trials (Figure 1b) [23]. Similar to the DNA coordination, cisplatin can also coordinate with carboxyl and amine groups that are widely present in polymeric NPs. Polymeric micelles, such as NC-6004 and AP5280 (HPMA-cisplatin conjugate), have also been under clinical investigation (Figure 1c,d) [11,18]. In addition to loading cisplatin into pre-formed NPs, cisplatin can also be engineered into nanomedicine through its coordination with polymers, which simultaneously enables NP formation and cisplatin loading. Representative examples include cisplatin-crosslinked polysaccharides [9,24], albumin [14,25] and DNA [26]. In addition to these organic NPs for cisplatin delivery (Figure 1e,f), inorganic NPs such as iron oxide and silica NPs have also been widely explored for their ability to deliver cisplatin (Figure 1g,h) [15,16,27]. For a comprehensive and detailed overview of cisplatin-based nanomedicine, interested readers are referred to specialized reviews for more detailed discussions [18,22,28].

## 3. Determination of Cisplatin Loaded in NPs

### 3.1. The Importance of Accurate Determination of Cisplatin in Nanomedicine

During the development of cisplatin nanomedicine, characterization of cisplatin is needed. Accurate quantification of cisplatin is crucial, as it directly influences the evaluation of drug loading efficiency, release profiles, and therapeutic performance. A precise measurement not only ensures reliable dosing and minimizes systemic toxicity, but also supports the reproducibility of experimental results. Moreover, consistent quantification is vital for comparing different nanoformulations and for meeting regulatory requirements during clinical translation. Because various nanomaterials have been employed for cisplatin delivery, they may introduce different interfering factors that hinder precise quantification. Inaccurate quantifications may result in safety risks, misleading release profiles, poor reproducibility, and regulatory setbacks.

### 3.2. The Main Strategies Used for Quantification of Cisplatin in NPs

Multiple techniques are available for cisplatin quantification [29,30,31]. Based on the detection principle applied, cisplatin quantification can be broadly classified into four different strategies, primarily based on cisplatin’s intrinsic physical properties, chemical composition, coordination derivatives, and electrochemical properties. Typical examples include: (1) spectrophotometric methods based on absorbance and high-performance liquid chromatography (HPLC), which rely on cisplatin’s polarity and hydrophobicity; (2) elemental analysis, such as platinum (Pt) determination using inductively coupled plasma mass spectrometry (ICP-MS); (3) cisplatin’s coordination with o-phenylenediamine (OPDA); and (4) electrochemical sensors. The encapsulation of cisplatin into NPs introduces additional interference factors into cisplatin determination, which depend on the detection technique, NP type, and solution environment. In this review, representative quantification strategies for cisplatin-loaded NPs are summarized, and the characteristics of each technique discussed, using cisplatin nanomedicine as an example.

#### 3.2.1. Spectrophotometric Method

In pure water, cisplatin demonstrates an absorbance peak around 301 nm [30,32]. Although the molar extinction coefficient of cisplatin is low [30,33], its UV absorbance can still be used to quantify cisplatin for NP characterization. As most NPs such as noble metal NPs, carbon nanomaterials, and micelles also demonstrate strong absorption in the UV region, a direct determination of cisplatin in NPs based on UV absorbance is challenging. Alternatively, cisplatin extracted or released from NPs can be quantified based on a spectrophotometric method, which can indirectly reflect cisplatin loading efficiency and release behavior under different conditions [34,35,36,37]. Typically, cisplatin in the NP supernatant is harvested and the absorbance then recorded using a UV/Vis spectrometer [38,39]. After referring to the standard curve of cisplatin absorbance, cisplatin content in NPs can be calculated. This spectrophotometric method for cisplatin determination is simple, fast, and cost-effective; however, its applications are also limited. Besides interference from NP absorbance, cisplatin absorbance can also be easily affected by its interaction with solvents (e.g., DMSO) or thiol-containing molecules (e.g., GSH), which are commonly found during the handling of cisplatin-loaded NPs for drug release evaluation [17,40,41]. Thus, when using the spectrophotometric method, the solution conditions for cisplatin determination should also be taken into consideration.

#### 3.2.2. HPLC Techniques

HPLC methods have been widely used to quantify cisplatin in solution, biological samples, and NP formulations [29,42]. In terms of principle, HPLC can efficiently separate cisplatin from a sample mixture based on polarity differences. With the assistance of coupled UV-Vis detectors, cisplatin contained in different samples can be determined with high sensitivity and specificity. The wavelengths of 210 nm and 301 nm can be utilized to detect the separated cisplatin [30,43]. Additionally, derivatization of cisplatin, such as using diethyldithiocarbamate (DDTC), can further increase detection sensitivity. Both post- and pre-column derivatization strategies have been used to generate the Pt(DDTC) complex, and this complex has been detected at 254 nm using a UV detector [44,45,46]. When HPLC is coupled with mass spectrometry (HPLC-MS), this technique combines the separation capability of HPLC and the precise identification of MS to accurately determine cisplatin and its derivatives. This not only offers the advantage of characterizing cisplatin metabolism within the body, but also allows differentiation of different cisplatin coordination complexes, even in the case of cisplatin-loaded NPs administered in vivo. [29]. Jin et al. reported that HPLC-MS could efficiently determine cisplatin loading efficiency within a self-assembled NP prepared from a folate derivative (FA-2-DG), and simultaneously confirmed the coordination structure of cisplatin and folate [47]. Compared with other analysis methods, HPLC-based strategies demonstrate a simultaneous quantification of different cisplatin species. However, the detection of cisplatin in NPs still requires pretreatment steps to disrupt the NPs or extract the cisplatin [46]. For example, the authors of one study found that cisplatin in magnetic NPs that were modified by folate-conjugated albumin needed to be released first by pepsin digestion; cisplatin in supernatant was then collected through centrifugation before the HPLC quantification [48].

#### 3.2.3. Quantitative Analysis of Pt Element

Given that each cisplatin molecule contains one Pt atom, direct quantification of Pt can accurately reflect the cisplatin content. Typical elemental analysis techniques, such as inductively coupled plasma mass spectrometry (ICP-MS), inductively coupled plasma optical emission spectrometry (ICP-OES), and atomic absorption spectrometry (AAS) [49,50], have been widely used to quantify metal-based drugs. Both ICP-MS and ICP-OES can simultaneously measure multiple elements, whereas AAS is only suitable for the measurement of a single element. With appropriate sample pretreatment, these three techniques are capable of measuring the cisplatin content in NP samples, demonstrating a detection sensitivity ranking of ICP-MS > ICP-OES > AAS. For example, ICP-MS can detect the metal ions at parts per trillion (ppt) levels, and thus is suitable for analyzing trace amounts of cisplatin within NPs, including quantification of cisplatin’s loading efficiency, as well as evaluation of cellular uptake, pharmacokinetics, and tissue distribution in cisplatin-loaded NPs [29,30,51,52]. A representative protocol for cisplatin determination using ICP-MS or ICP-OES involves acidic digestion of NPs, followed by dilution and neutralization of the resulting solution prior to platinum measurement [53,54,55]. After this, cisplatin concentration can be calculated according to a platinum standard curve derived from different concentrations. Elemental determination techniques, such as ICP-MS, are intricate, as they require the transformation of cisplatin-containing NPs into Pt ions with the assistance of strong acids (e.g., aqua regia, nitric acid). Consequently, the whole process is relatively time-consuming and expensive, and requires specialized training. However, this elemental analysis strategy represents the most powerful technique to specifically determine Pt content with high sensitivity. It should be noted that Pt measurements cannot differentiate the different cisplatin species present in a sample. To give an extreme example, when cisplatin coexists with platinum NPs [56,57], a direct ICP-MS determination of the carriers would overestimate the cisplatin quantity. In this case, in order to accurately determine cisplatin using elemental analysis, extraction of it from the carriers would still be needed.

#### 3.2.4. Spectrophotometric Determination Based on Cisplatin’s Derivatizing Reaction

While spectrophotometric measurement of cisplatin is limited due to its UV-Vis absorption, its coordination with chromogenic agents can generate a distinct color that is easily detected using a spectrophotometer or a widely available plate reader. As mentioned above, the derivatization of cisplatin with DDTC has been used for HPLC with a UV detector after extraction or separation of cisplatin [45,46]. However, accurately measuring the absorbance of Pt(DDTC) derived from NPs without pretreatment is highly challenging due to strong absorbance interference from solvents, NPs and other components (e.g., a co-encapsulated drug within the NPs). In contrast, the coordination between cisplatin and o-phenylenediamine (OPDA) generates an absorption peak around 705 nm, which falls within the near-infrared (NIR) window and thus enables rapid determination of cisplatin in NP samples. Golla and Ayres were the pioneers in utilizing OPDA derivatization to spectrophotometrically determine both Pt (IV) and Pt (II) over 50 years ago [58]. They found that heating the mixture of a Pt compound with OPDA at 100 °C rapidly produced a light-blue product, which could be dissolved in dimethylformamide (DMF) and remained stable for 24 h. The cisplatin-OPDA coordination product was then identified and shown to be linearly correlated with cisplatin content when excess OPDA was present [59,60]. As the absorbance peak of cisplatin-OPDA adducts is far from that of native cisplatin and most absorbing species present in biological samples, this method has been used to determine cisplatin in water [61], tablets [60], and urine [59]. Due to the simplicity, low cost, and precision of the OPDA method, it has been widely used to determine cisplatin loading efficiency and the release behavior of various NPs, including liposomes [62,63], micelles [64,65], organic NPs [66,67], polysaccharide NPs [68,69], DNA NPs [70,71], and silica NPs [72,73].

**Figure 2:**
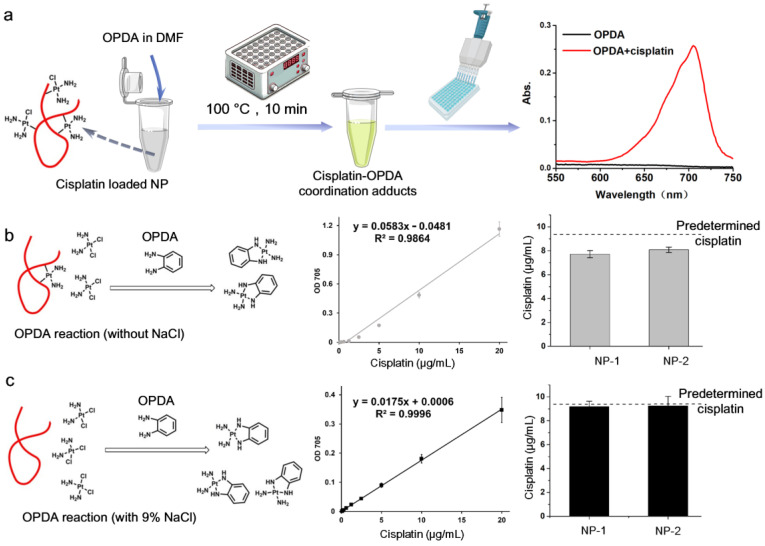
The OPDA method to determine cisplatin loaded in NPs. (**a**) A representative protocol for the OPDA method for cisplatin determination. (**b**) The OPDA reaction without the presence of chloride ions. Without chloride interference, the tight binding between cisplatin and NPs leads to an underestimation of the cisplatin quantity determined by the OPDA reaction. (**c**) The introduction of NaCl into the OPDA reaction increases the cisplatin determination capability.

More importantly, OPDA can be directly used to measure the cisplatin in NPs, as the combination of added DMF and high temperature helps to disrupt NPs, without the requirement for complicated pretreatment (Figure 2a). Introduction of chloride into the reaction medium was found to increase the detachment of cisplatin from a sample [13,59], allowing a complete determination of loaded cisplatin, compared with OPDA quantification conducted without chloride (Figure 2b,c). Although adding a chloride such as NaCl partially inhibits the cisplatin-OPDA production, it can improve detection capability through a one-step extraction of cisplatin tightly bound to NPs [13]. Generally, OPDA derivatization depends on the pH, temperature, salt, solvent, etc. [59,60,61]. For the characterization of cisplatin-loaded NP, the solution environment may vary significantly between different NP types and experimental purposes. For example, to evaluate pH-responsive release behavior of cisplatin from NPs, buffers of different pH with or without salts may be used. In this case, a cisplatin standard curve in the buffer solution corresponding to that used for the cisplatin release experiment is necessary to precisely determine the cisplatin content. While the OPDA method strictly depends on coordination with cisplatin, it may also efficiently reflect the bioactive form of cisplatin. It should be noted that this OPDA method may be affected by interference from thiol/sulfa-containing environment. Currently, there is no standardized protocol for the OPDA method to determine cisplatin in NPs. Standardization of this analysis would enable consistent comparison of cisplatin characterization across different studies.

#### 3.2.5. Electrochemical Determination

While the electrolysis of a platinum electrode led to the discovery of cisplatin as an anticancer agent, the electrochemical activity of platinum-based drugs provides an effective option for cisplatin quantification through electroanalytical methods [74,75,76]. Electrochemical biosensors demonstrate unique advantages for measuring cisplatin, including ease of operation, rapid detection and low cost [31,77,78]. The platinum–DNA binding complex can be easily detected by voltammetry using a mercury electrode, and similar strategies have also been widely used to detect cisplatin in aqueous solutions, biological samples, and in vivo environments [79,80,81,82]. A hanging mercury drop electrode (HMDE) modified by EDTA-metallothionein (MT) can selectively bind Pt(II) from DNA adducts via MT’s thiol groups, enabling sensitive detection of cisplatin-DNA [80]. Wu et al. reported an electrochemical sensor constructed using a DNA probe, which was simultaneously conjugated with thiol and methylene blue for cisplatin determination [83,84]. The binding between platinum and DNA altered the DNA conformation, changed the probe coverage, and thus acted as a signal-off or signal-on sensor for cisplatin. More recently, research efforts have focused on nanomaterial-based electrochemical sensors for signal amplification; these have demonstrated high sensitivity and selectivity and thus potentially enable real-time sensing of cisplatin. These strategies include electrodes modified with carbon nanotubes, bismuth NPs, graphene, and Pd-Fe NPs [85,86,87,88]. Overall, the quantification range of electrochemical biosensors for cisplatin is highly dependent on the electrode materials and surface modifications employed. For example, a bismuth NP/graphene-modified glassy carbon electrode demonstrated a limit of detection (LOD) of 4.4 μM for cisplatin, with a linear range of 6.0 to 180 μM [87]. This electrochemical sensor also showed performance comparable to that of the HPLC-UV method when detecting cisplatin in serum samples, indicating its potential for clinical and biomedical applications.

**Figure 3:**
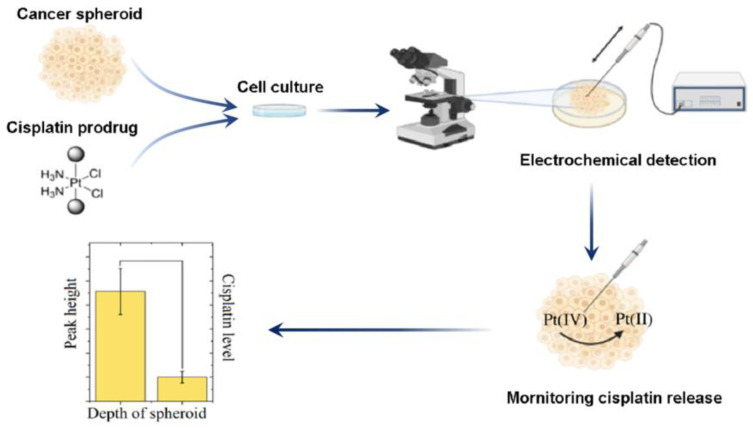
Real-time monitoring of cisplatin release in a living cancer spheroid using electrochemical sensors. Adapted from Ref. [91] with permission from American Chemical Society.

While electrochemical methods are less likely to detect cisplatin loaded in NPs, they can be used to monitor the released cisplatin as well as its coordination with DNA in real time. De Miguel et al. reported that the voltammetric response of released cisplatin could be used to monitor the release behavior of cisplatin that was loaded in a poly (γ-benzyl-L-glutamate)-poly(glutamic acid) block polymer (CDDP-PBLG-b-PGlu) NP [89]. Moreover, electrochemical sensors can also be adapted to verify the coordination between released cisplatin and DNA, providing evidence that the cisplatin delivered by NPs remains biologically functional [90]. Krasnovskaya et al. successfully employed Pt-coated nanoelectrodes to monitor the release of cisplatin from a Pt (IV) prodrug within a living tumor spheroid [91,92] (Figure 3). Compared with other strategies, the application of electrochemical techniques for cisplatin quantification in NPs is less common. However, it can be used to monitor cisplatin activities, even in living organisms, and thus may provide more insights into the therapeutic mechanisms of cisplatin-based nanomedicine.

## 4. Conclusions and Perspectives

Cisplatin-loaded NPs have been widely explored as anticancer nanomedicines for various cancers. During the production of cisplatin nanomedicine, the evaluation of cisplatin loading, stability, and release behavior is essential for NP characterization. Based on the physicochemical properties of cisplatin, this review summarizes representative characterization strategies for cisplatin loaded in NPs. Specifically, representative analytical methods such as UV-Vis, HPLC, ICP-MS, OPDA, and electrochemical techniques are discussed (Table 1). The separation of cisplatin from NPs is useful for indirectly calculating drug loading, although it requires additional pretreatment steps. The extracted cisplatin can be analyzed using all these detection techniques. For the direct determination of cisplatin loaded in NPs, an appropriate quantification strategy should be selected according to the type of NPs, the co-loaded compounds, and the solvent environment, among other factors. Overall, while cisplatin detection methods with higher specificity may require more time and higher costs, simpler methods offer faster analysis at lower expense. For example, HPLC-MS and ICP-MS can detect cisplatin species or Pt content with high specificity and sensitivity, respectively, but both require sophisticated instrumentation, resulting in higher costs. UV-Vis detection based on cisplatin absorbance is simple and inexpensive; however, its application is limited because UV signals can be affected by interference from NPs, solvents, and other factors. Given this, the OPDA method efficiently bridges the gap between direct UV absorbance measurement, HPLC, and elemental analysis. The OPDA method enables rapid quantification of cisplatin in various NPs, with good sensitivity and low cost, and can be performed using a standard plate reader or UV photometer. Most importantly, the emerging electrochemical detection of cisplatin may be used to potentially monitor both cisplatin release and its binding with DNA in living cells, providing a promising strategy for characterizing cisplatin-loaded NPs during the drug delivery process. Future development of novel techniques or integrated multi-method approaches will enable more accurate and comprehensive quantification of cisplatin in NPs.

## Figures and Tables

**Table 1:** Summary of the main methods for cisplatin quantification in nanomedicine.

Methods	Principle	Measurement Time	Pros	Cons
Spectrophotometric method	UV-Vis absorbance	<10 min	Simple, cheap	Low sensitivity and specificity
HPLC/HPLC-MS	Chromatographic separation and detection	~20–60 min	High specificity, structure determination (with MS)	Sample pretreatment, sophisticated instrumentation
ICP-MS/OES	Elemental analysis of Pt	~10–30 min	Excellent sensitivity and accuracy	Sample pretreatment, sophisticated instrumentation, detection of the ion form of Pt
OPDA method	Derivatization of cisplatin with OPDA	~20 min	Simple, cheap, user-friendly	Interference by sulfurs/thiols
Electrochemical determination	Redox activity of Pt (II)	~10 min	Ease of operation, cheap, rapid detection	Sample pretreatment, interference by other electroactive species, less used for cisplatin nanomedicine

## Data Availability

Not applicable.
